# Association between accelerometer-measured light-intensity physical activity and tumor regression for male patients with esophageal cancer receiving neoadjuvant therapy: a retrospective cohort study

**DOI:** 10.1007/s10388-025-01108-9

**Published:** 2025-02-04

**Authors:** Tomohiro Ikeda, Kazuhiro Noma, Masanori Konuma, Naoaki Maeda, Shunsuke Tanabe, Takayoshi Kawabata, Masashi Kanai, Masanori Hamada, Toshiyoshi Fujiwara, Toshifumi Ozaki

**Affiliations:** 1 Department of Rehabilitation Medicine, Okayama University Hospital, 2-5-1 Shikatacho, Kita-ku, Okayama, 700-8558 Japan; 2 Department of Gastroenterological Surgery, Graduate School of Medicine, Dentistry and Pharmaceutical Sciences, Okayama University, 2-5-1 Shikatacho, Kita-ku, Okayama, 700-8558 Japan; 3 Center for Esophageal Disease, Okayama University Hospital, 2-5-1 Shikatacho, Kita-ku, Okayama, 700-8558 Japan; 4 Department of Pharmacy, Okayama University Hospital, 2-5-1 Shikatacho, Kita-ku, Okayama, 700-8558 Japan; 5 Institute of Transdisciplinary Sciences for Innovation, Kanazawa University, Ishikawa, Japan; 6 Department of Orthopaedic Surgery, Okayama University Hospital, 2-5-1 Shikatacho, Kitaku, Okayama 700-8558 Japan

**Keywords:** Prehabilitation, Neoadjuvant therapy, Esophageal cancer, Physical activity, Tumor regression

## Abstract

**Background:**

Physical activity has the potential to promote tumor regression in patients with esophageal cancer receiving neoadjuvant chemotherapy (NAC); however, the benefits of light-intensity physical activity (LIPA) are unclear. This study aimed to investigate the impact of LIPA on tumor regression in male patients with esophageal cancer during NAC and its optimal cutoff value.

**Methods:**

This retrospective single-center observational study included all male patients who underwent NAC or curative esophagectomy. We assessed the physical activity of patients using an accelerometer and calculated the time spent on LIPA. Tumor regression was defined as grade ≥ 1b according to the Japanese classification of esophageal cancer. The impact of LIPA on tumor regression was analyzed using multivariate analysis, and the optimal cutoff value was identified using the receiver operating characteristic curve.

**Results:**

Sixty-nine male patients with esophageal cancer who underwent NAC were analyzed. The mean age was 68 years, mean body mass index was 22.4, and 80% of the patients were diagnosed with clinical stage 3 or 4 disease. Every extra 30-min increase in LIPA during the treatment phase was associated with tumor regression (adjusted OR 1.41 [1.02–2.04]). The optimal cutoff value of LIPA was 156.11 min/day, and patients with rich LIPA (≥ 156.11 min/day) were less likely to suffer from anorexia and malnutrition during NAC.

**Conclusion:**

This study demonstrated that LIPA during NAC has a potential of promoting tumor regression with a cutoff value of 156.5 min/day. Further clinical research is required to determine the prognostic benefits of LIPA in patients receiving NAC.

**Supplementary Information:**

The online version contains supplementary material available at 10.1007/s10388-025-01108-9.

## Introduction

Esophageal cancer is a common and lethal tumor [1]. The standard treatment for locally advanced esophageal cancer is neoadjuvant chemotherapy (NAC) followed by surgery [2]. Patients with esophageal cancer that is down-staged by NAC benefit from lower rates of local and systemic recurrence [3]. In addition, NAC improves dysphagia, minimizes surgical invasion by tumor regression. Therefore, there is a strong need for supportive care to enhance tumor regression with NAC.

Studies regarding the effects of physical activity on tumor regression are developing [4]. Fundamental research has demonstrated that physical activity may prevent tumor growth by alleviating tumor hypoxia, which is a risk factor for tumor growth, and activating tumor immunity in the tumor microenvironment [5]. Clinical trials similarly demonstrate the potential of moderate- or high-intensity physical activity as a supportive therapy to promote tumor regression in patients with esophageal and other cancers [6–8] However, patients with advanced esophageal cancer receiving chemotherapy often have difficulty performing moderate- or high-intensity exercise because of adverse events [9]. Therefore, there is a significant gap between evidence and clinical practice.

Naito et al. [10] reported that adherence to light-intensity physical activity (LIPA), which is included in multimodal interventions, is well accepted. In addition, its health benefits have been previously reported [11]. Although LIPA is acceptable and potentially beneficial, its effect on tumor regression in patients with esophageal cancer receiving NAC remains unknown. Hence, this study aimed to investigate the impact of LIPA on tumor regression in patients with esophageal cancer during NAC and its optimal cutoff value.

## Materials and methods

### Design and participants

This was a retrospective cohort single-center study. All participants underwent curative esophagectomy, including cervical, thoracic, and abdominal surgeries, at the Okayama University Hospital in Japan between October 2018 and December 2022. Study size was determined to be feasible. The inclusion criteria were as follows: male patients, completion of one–three courses of docetaxel/cisplatin/5-fluorouracil (DCF) on a standardized schedule, receipt of multimodal support from the perioperative team during NAC, receiving physical therapy from pre-NAC to surgery and the ability to ambulate without assistance. Two courses of DCF at 100% dose were administered as the standard treatment at the hospital where this study was conducted. In cases of renal dysfunction or reduced performance status, the number of treatment courses was reduced, or the dosing intensity was adjusted to 80%. Three courses of DCF were also an option to enhance treatment effectiveness. The exclusion criteria were missing physical activity data during NAC, diagnosis of dementia, and treatment other than DCF. This study was approved by the Ethics Committee of the Okayama University Hospital (approval number 2306-029) and was performed in accordance with the Declaration of Helsinki. Because of the nature of this study, an opt-out consent process was used.

### Tumor regression

Tumor regression was graded by a pathologist blinded to information on physical activity according to the Japanese Classification of Esophageal Cancer as follows: grade 0, no recognizable cytological or histological therapeutic effect; grade 1a, viable cancer cells accounting for two-thirds of the tumor; grade 1b, viable cancer cells accounting for one-third or more, but less than two-thirds of the tumor; grade 2, viable cancer cells accounting for less than one-third of the tumor; and grade 3, no evidence of viable cancer cells [12].

### Physical activity

The physical activity of the patients was assessed using the Active style Pro HJA-750IT (Omron Healthcare, Kyoto, Japan), which estimates metabolic equivalents (METs) every 10 s based on composite accelerations measured with a built-in tri-axial accelerometer. The algorithm and its validity are described in previous reports [13–15]. To be eligible for the analysis, the participants were required to wear the accelerometer for at least 4 days with at least 10 h of wear time each day [16]. Non-wear time was defined as at least 60 consecutive minutes of < 0.9 METs, with an allowance for up to 2 min of some limited movement (≤ 1.0 METs) within these periods [16]. The daily average time spent in sedentary behavior (≤ 1.5 METs), LIPA (> 1.5 to < 3.0 METs), and moderate to vigorous physical activity (≥ 3.0 METs) [16]. Physical activity was assessed during each treatment phase (day 1–14) and intermission phase (day 15–28).

### Data collection

We obtained the information from medical records, which included age, sex, clinical and pathological stage according to the UICC-TNM classification (8th edition) [17], the maximum length, the orthogonally oriented maximum width of the tumor based on pre-and post-NAC computed tomography images according to the Japanese Classification of Esophageal Cancer, 12th Edition [18], NAC information, body mass index (BMI), malnutrition defined as geriatric nutritional risk index < 98 [19], neutrophil/lymphocyte ratio [20], C-reactive protein [21], Charlson comorbidity index (CCI), adverse events during NAC, postoperative complications based on the Clavien–Dindo classification (grade ≥ 2), and length of intensive care unit stay. Adverse events were defined as grade 3 or higher based on the Common Terminology Criteria for Adverse Events version 5.0, which was based on the JCOG guidelines.

### Statistical analysis

All statistical analyses were performed using the R software (version 4.0.2). Statistical significance was set at *p* < 0.05 for all analyses. First, to evaluate the association between LIPA and tumor regression, we used three logistic regression models: the Crude Model, Adjusted Model 1, and Adjusted Model 2. Odds ratio (OR) and 95% confidence interval (CI) were calculated for each model. Tumor regression, the outcome variable in these analyses, was defined as grade ≥ 1b (necrotic or fibrotic changes observed in more than 1/3 of the tumors) [22]. The increase in LIPA every extra 30 min, treated as an exposure factor, was included as an explanatory variable. This unit was selected to facilitate clinical interpretation and to provide practical insights for patient communication. For the Crude Model, a univariate logistic regression analysis was conducted to assess the direct association between tumor regression and LIPA. For the Adjusted Model 1, multivariate logistic regression was performed while adjusting for the following confounding factors: age, BMI, clinical stage at diagnosis, CCI (grade ≥ 2) [23], and wearing time of the accelerometer. For Adjustment Model 2, regimens (standard, reduced, or enhanced) were added to the confounding factors in Adjustment Model 1.

Second, to set the cutoff value for LIPA, a receiver operating characteristic (ROC) curve was constructed. Patients were categorized into two groups (Poor-LIPA and Rich-LIPA) based on the cutoff value established by the ROC curve. The association between LIPA and tumor regression was subsequently analyzed by incorporating the categorical variable Rich-LIPA as an exposure variable into the three logistic regression models previously described (Crude Model, Adjusted Model 1, and Adjusted Model 2).

Third, the clinicopathological characteristics and postoperative clinical outcomes of the two groups (Poor-LIPA and Rich-LIPA groups) were assessed. To analyze the differences between the two groups, we checked the normality of the data and performed the Wilcoxon rank-sum test, t-test, chi-square test of independence, and Fisher’s exact test.

The association between tumor regression and LIPA was reanalyzed as a sensitivity analysis using an alternative cutoff value of grade ≥ 2, defined as necrotic or fibrotic changes observed in more than 2/3 of the tumor.

## Results

### Clinical characteristics before NAC

**Fig. 1:**
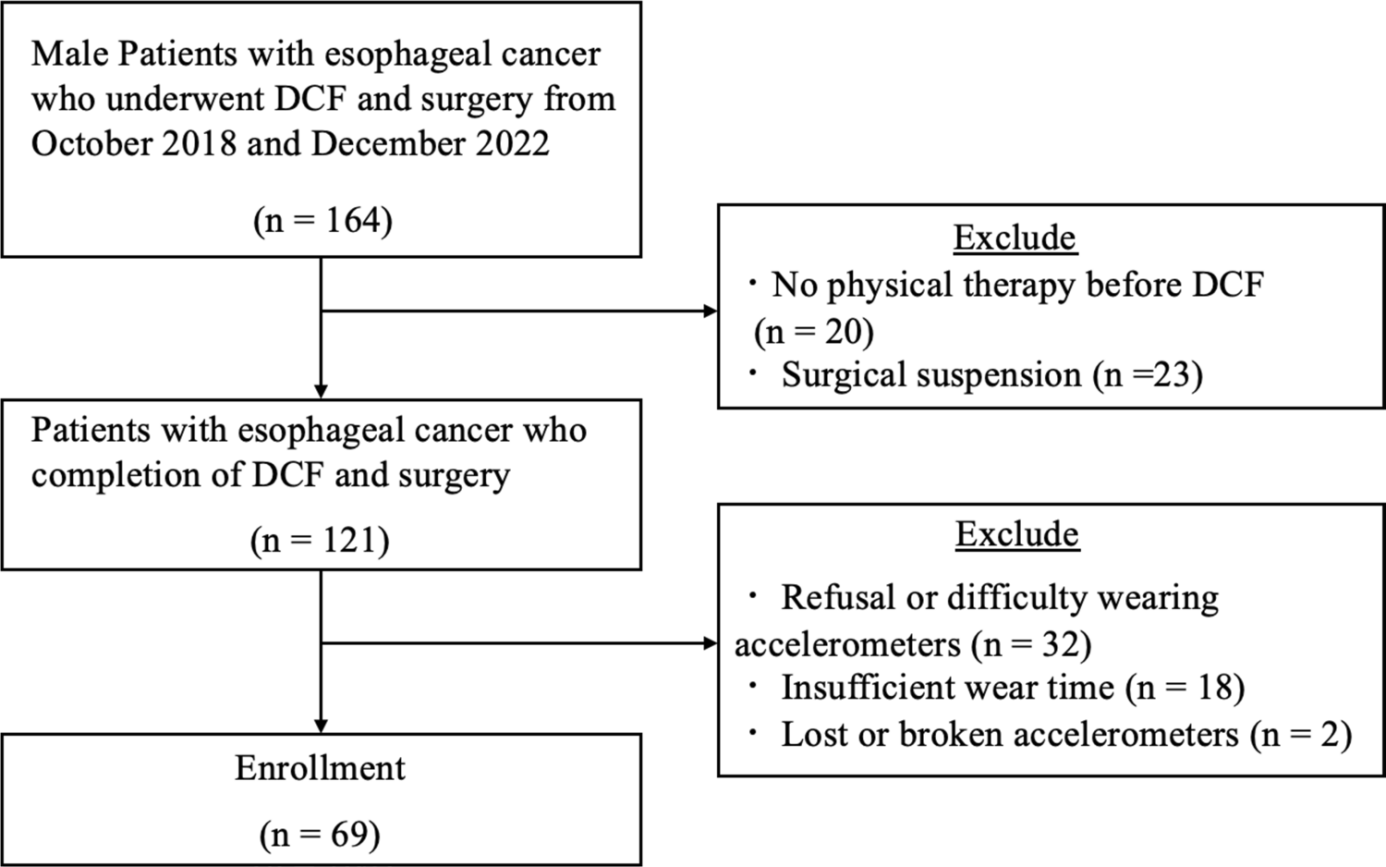
Flow diagram of the enrolled patients. DCF, docetaxel/cisplatin/5-fluorouracil

Of the 164 patients initially included, 95 were excluded for the following reasons: no physical therapy before NAC (n = 20), surgical suspension (n = 23), refusal or difficulty in wearing the accelerometers (n = 32), insufficient wear time (n = 18), and lost or broken accelerometers (n = 2). Finally, 69 male patients were analyzed (Fig. 1). The clinical characteristics of the participants before NAC were as follows: mean age, 68 years; mean BMI, 22.4; 80% had clinical stage 3 or 4 disease; and 77% were diagnosed with thoracic esophageal cancer and 90% with squamous cell carcinoma (SCC). In addition, 58.2% of patients required a soft diet or feeding tube (Table 1).

**Table 1:** Clinicopathological characteristics of the two groups before neoadjuvant chemotherapy

Characteristic		Overall N = 69
Age (years)		68 (63, 72)
BMI (kg/m^2^)		22.4 (19.8, 24.4)
Clinical stage	III–IV	55 (80%)
cT	≥ 3	60 (87%)
cN	≥ 1	56 (81%)
cM	= 1	0 (0%)
Location^a^		
	Ae	9 (13%)
	Ce	7 (10%)
	Te	53 (77%)
Type^b^		
	SCC	62 (90%)
	AC	7 (10%)
PS^c^	≥ 1	6 (8.7%)
CCI^d^	≥ 1	29 (42%)
Dietary	Normal	29 (42%)
	Soft	35 (51%)
	Tube	5 (7.2%)
Brinkman index^e^		705 (350, 980)
Baseline laboratory test result		
Albumin (g/dl)		4.1 (3.6, 4.3)
Neutrophils (× 10^3^/μl)		4.4 (3.1, 5.2)
Lymphocytes (× 10^3^/μl)		1.5 (1.2, 2.1)
C-reactive protein (mg/dl)		0.2 (0.1, 0.4)
Hemoglobin (mg/dl)		14.0 (13.1, 14.8)
Malnutrition^f^		23 (33%)
NLR^g^		2.6 (1.8, 3.8)

n (%); median (IQR)

Fisher's exact test; Wilcoxon rank sum test; Pearson's Chi-squared test

^a^Ae abdominal esophagus, Te thoracic esophagus, Ce cervical esophagus,

^b^SCC squamous cell carcinoma, AC adenocarcinoma,

^c^PS performance status

^d^CCI Charlson comorbidity index

^e^Brinkman index; multiplying the average number of cigarettes smoked per day by the number of years the person has smoked

^f^Malnutrition GNRI (geriatric nutritional risk index) score < 98

^g^NLR neutrophil-to-lymphocyte ratio (neutrophils/lymphocytes)

### The impact of every extra 30-min in LIPA on the tumor regression

Every extra 30-min in LIPA during the treatment phase was associated with the tumor regression defined as grade ≥ 1b for the Crude Model (OR 1.42, 95% CI 1.06–1.99, *p* = 0.029), Adjusted Model 1 (OR 1.41, 95% CI 1.03–2.03, *p* = 0.042), and Adjusted Model 2 (OR 1.41, 95% CI 1.02–2.04, *p* = 0.049) (Table 2). However, the LIPA time in the treatment phase was not significantly associated with the tumor regression that was defined as grade ≥ 2 for the Crude Model (OR 1.28, 95% CI:0.97–1.73, *p* = 0.092), Adjusted Model 1 (OR 1.25, 95% CI 0.94–1.70, *p* = 0.137), and Adjusted Model 2 (OR 1.24, 95% CI 0.93–1.70, *p* = 0.149) (Online Resource 1). The LIPA during the intermission phase was not significantly associated with tumor regression.

**Table 2:** The association between every extra 30-min of LIPA and tumor regression (grade ≥ 1b)

	Crude Model^a^		Adjusted Model 1^b^		Adjusted Model 2^c^	
	OR^d^ (95%CI^e^)	p-value	OR (95%CI)	p-value	OR (95%CI)	p-value
LIPA in TR^f^ (30 min/day)	1.42 (1.06, 1.99)	0.029*	1.41 (1.03, 2.03)	0.042*	1.41 (1.02, 2.04)	0.049*
LIPA in INT^g^ (30 min/day)	1.01 (0.84, 1.22)	0.924	1.04 (0.83, 1.30)	0.737	1.01 (0.80, 1.26)	0.964

The results of regression analysis are shown for the effect of LIPA measured by accelerometer on tumor regression (grade ≥ 1b: necrotic or fibrotic changes are observed in more than 1/3 of the tumors)

*LIPA* light intensity physical activity

*Statistically significant difference (p < 0.05)

^a^Crude Model univariate analysis

^b^Adjusted Model 1 adjusted for age, BMI, stage, CCI, and wearing time of the accelerometer

^c^Adjusted Model 2 adjusted for age, BMI, stage, CCI, wearing time of the accelerometer, and regimen (standard, reduced, or enhanced)

^d^OR odds ratio

^e^95% CI 95% confidence interval

^f^LIPA in TR every extra 30-min of light intensity physical activity in the treatment phase

^g^LIPA in INT every extra 30-min of light intensity physical activity in the intermission phase

### Cutoff value for LIPA on tumor regression

**Fig. 2:**
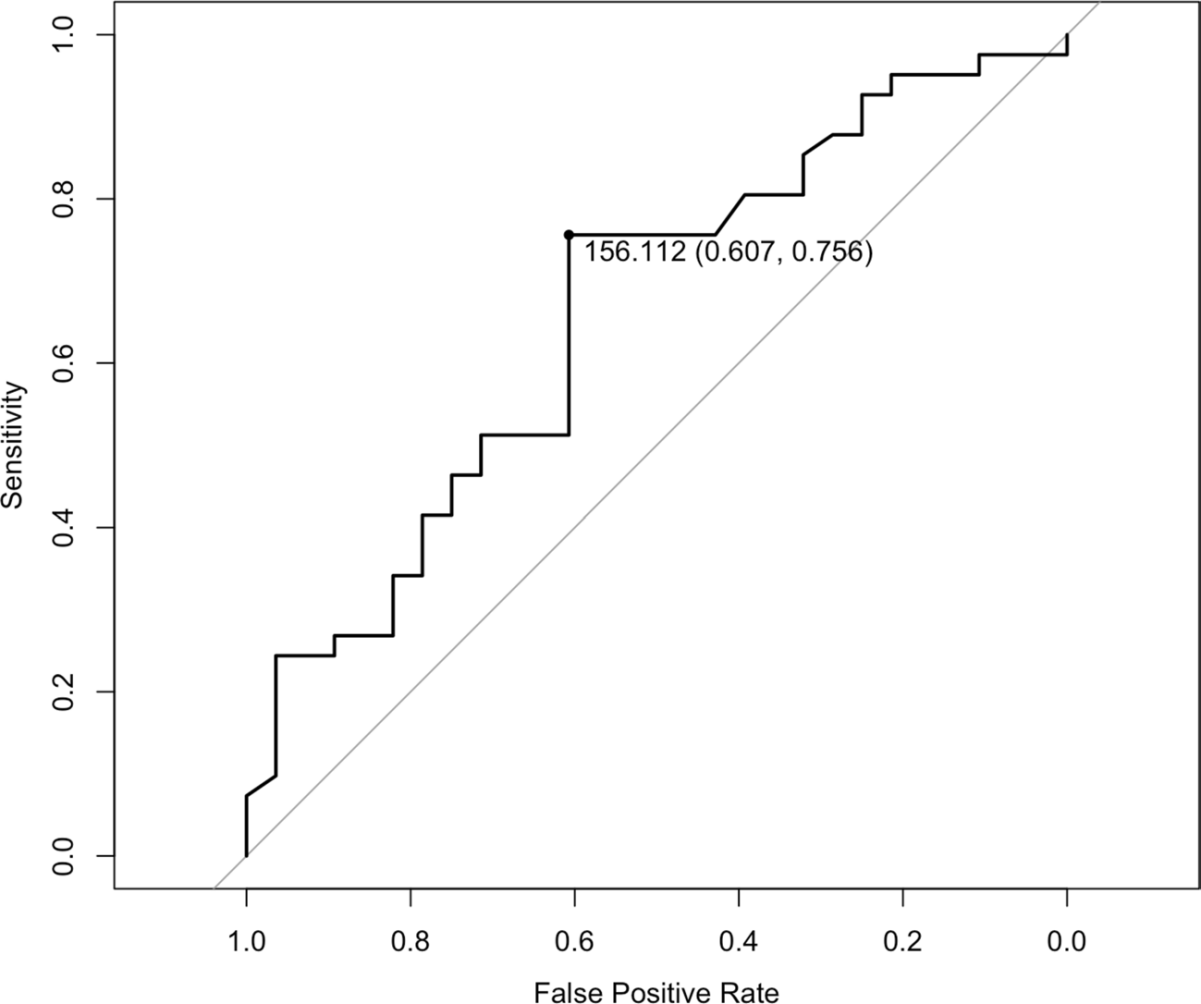
The ROC curve and cutoff point of LIPA in the intermission phase for tumor regression. The cutoff value defined using the ROC curves for LIPA during the treatment phase was 156.11 min/day (area under the curve, 0.660; sensitivity, 0.607; specificity, 0.756). ROC, receiver operating characteristic; LIPA, light-intensity physical activity

The cutoff value, which was defined using ROC curves, for LIPA during the treatment phase was 156.11 min/day (area under curve: 0.660, sensitivity: 0.607, specificity: 0.756; Fig. 2). Patients were classified as Rich-LIPA (≥ 156.11 min/day) or Poor-LIPA (< 156.11 min/day). The median (interquartile range) of LIPA in the treatment phase was 134 (116–146) min/day in the Poor-LIPA group and 196 (176–236) min/day in the Rich-LIPA group. Grade ≥ 1b tumor regression was 39% in the Poor-LIPA group and 73% in the Rich-LIPA group, and grade ≥ 2 tumor regression was 18% and 54%, respectively. In the Rich-LIPA group, the maximum tumor length was significantly reduced after NAC (− 16 [− 25, − 8] mm vs − 6 [− 17, − 4] mm, *p* = 0.009). The clinical lymph node downstaging rate was 7.1% and 24% in the Poor- and Rich-LIPA groups, respectively (Table 3).

**Table 3:** Physical activity and treatment response during neoadjuvant chemotherapy

Characteristic		Overall N = 69	Rich-LIPA^a^ N = 41	Poor-LIPA^b^ N = 28	*p*-value
Physical activity indexes					
Treatment phase^c^					
SB^d^ (min/day)		705 (537, 890)	588 (509, 817)	763 (630, 995)	0.006*
LIPA^e^ (min/day)	Total^h^	169 (141, 210)	196 (176, 236)	134 (116, 146)	< 0.001*
	1st^i^	137 (110, 193)	177 (146, 202)	102 (90, 123)	< 0.001*
	2nd^j^	210 (159, 241)	239 (212, 253)	156 (143, 165)	< 0.001*
MVPA^f^ (min/day)		19 (8, 26)	21 (9, 25)	10 (6, 26)	0.156
Wearing time^g.^ (min/day)		848 (736, 1090)	824 (696, 1082)	903 (784, 1134)	0.119
Wearing days^g^ (min/day)		19 (11, 25)	19 (11, 24)	20 (11, 25)	0.569
Intermission phase. (NA = 6)					
SB (min/day)		511 (439, 636)	506 (390, 632)	514 (469, 639)	0.555
LIPA (min/day)	Total	213 (174, 276)	221 (180, 280)	210 (172, 258)	0.537
	1st	194 (156, 251)	199 (156, 253)	191 (158, 219)	0.574
	2nd	241 (189, 308)	250 (193, 314)	230 (186, 299)	0.841
MVPA (min/day)		33 (21, 58)	33 (24, 67)	30 (18, 53)	0.158
Wearing time (min/day)		755 (691, 902)	752 (685, 928)	773 (721, 874)	0.939
Wearing days (min/day)		20 (11, 29)	22 (12, 32)	18 (11, 25)	0.184
Treatment response indexes					
Pathological tumor regression					0.009*
Grade 1a		28 (41%)	11 (27%)	17 (61%)	
Grade 1b		13 (19%)	8 (20%)	5 (18%)	
Grade 2		24 (35%)	20 (49%)	4 (14%)	
Grade 3		4 (5.8%)	2 (4.9%)	2 (7.1%)	
Grade ≥ 1b		41 (59%)	30 (73%)	11 (39%)	0.005*
Grade ≥ 2		27 (39%)	22 (54%)	5 (18%)	0.003*
Clinical downstaging					
T-downstaging		13 (19%)	8 (20%)	5 (18%)	0.863
N-downstaging		12 (17%)	10 (24%)	2 (7.1%)	0.104
Tumor size^k^					
Maximum length (mm)	Pre^l^	28 (23, 33)	29 (24, 37)	26 (21, 31)	0.031*
	Post^m^	17 (0, 21)	16 (0, 21)	17 (9, 22)	0.466
	Δ^n^	− 13 (− 24, − 5)	− 16 (− 25, − 8)	− 6 (− 17, − 4)	0.009*
Maximum width (mm)	Pre	18.1 (13.8, 22.8)	19.9 (14.2, 23.8)	17.0 (12.5, 22.2)	0.229
	Post	8 (0, 12)	8 (0, 13)	10 (5, 12)	0.328
	Δ	− 8 (− 16, − 5)	− 10 (− 19, − 5)	− 6 (− 9, − 3)	0.034*

n (%); median (IQR)

Fisher's exact test; Wilcoxon rank sum test; Pearson's Chi-squared test

*LIPA* light intensity physical activity

*Significant difference in the two groups (p < 0.05)

^a^Rich-LIPA patients whose LIPA in the treatment phase was ≥ 156.11 min/day

^b^Poor-LIPA patients whose LIPA in the treatment phase was < 156.11 min/day

^c^Treatment phase 2 weeks from the first day of chemotherapy

^d^SB sedentary behavior (≤ 1.5 METs)

^e^LIPA light intensity physical activity

^f^MVPA moderate to vigorous physical activity (≥ 3.0 to < 6.0 METs)

^g^Wearing time/days wearing days average hours and total days of accelerometer wear

^h^Total 1st to 2nd/3rd course of neoadjuvant chemotherapy

^i^1st 1st course of neoadjuvant chemotherapy

^j^2nd- 2nd or 3rd course of neoadjuvant chemotherapy

^k^Tumor size the maximum length, orthogonally oriented maximum width of the tumor based on computed tomography images according to Japanese Classification of Esophageal Cancer, 12th Edition

^l^Pre Before neoadjuvant chemotherapy

^m^Post After neoadjuvant chemotherapy

^n^Δ Changes pre- and post-neoadjuvant chemotherapy

### The impact of the Rich-LIPA group on the tumor regression

Rich-LIPA in the treatment phase is associated with the tumor regression that was defined as grade ≥ 1b for the Crude Model (OR 4.21, 95% CI 1.54–12.2, *p* = 0.006), Adjusted Model 1 (OR 4.05, 95% CI 1.43–12.2, *p* = 0.010), and Adjusted Model 2 (OR 4.37, 95% CI 1.47–14.1, *p* = 0.010) (Table 4). In addition, the Rich-LIPA in the treatment phase was associated with the tumor regression that was defined as grade ≥ 2 for the Crude Model (OR 5.33, 95% CI:1.80–18.4, *p* = 0.004), Adjusted Model 1 (OR 4.96, 95% CI 1.64–17.4, *p* = 0.007), and Adjusted Model 2 (OR 5.38, 95% CI 1.69–20.1, *p* = 0.007) (Online Resource 2). The LIPA in the intermission phase was not significantly associated with tumor regression.

**Table 4:** The association between Rich-LIPA and tumor regression (grade ≥ 1b)

	Crude Model^a^		Adjusted Model 1^b^		Adjusted Model 2^c^	
	OR^d^ (95%CI^e^)	p-value	OR (95%CI)	p-value	OR (95%CI)	p-value
Rich-LIPA in TR^f^	4.21 (1.54–12.2)	0.006*	4.05 (1.43–12.2)	0.010*	4.37 (1.47–14.1)	0.010*
Rich-LIPA in INT^g^	1.73 (0.61–5.02)	0.305	1.96 (0.61–6.54)	0.261	1.83 (0.55–6.37)	0.326

The results of regression analysis are shown for the effect of light intensity physical activity measured by accelerometer on tumor regression (grade 1b ≤ : necrotic or fibrotic changes are observed in more than 1/3 of the tumors)

Rich-LIPA patients whose LIPA in the treatment phase was ≥ 156.11 min/day

*LIPA* light intensity physical activity

*Statistically significant difference (p < 0.05)

^a^Crude Model univariate analysis

^b^Adjusted Model 1 adjusted for age, BMI, stage, CCI, and wearing time of the accelerometer

^c^Adjusted Model 2 adjusted for age, BMI, stage, CCI, wearing time of the accelerometer, and regimen (standard, reduced, or enhanced)

^d^OR odds ratio

^e^95% CI 95% confidence interval

^f^Rich-LIPA in TR patients whose light intensity physical activity in the treatment phase was > 156.11 min/day

^g^Rich-LIPA in INT patients whose light intensity physical activity in the intermission phase was > 191.31 min/day

### Clinical characteristics during NAC of the Rich- and Poor-LIPA groups

Patients who received standard treatment (DCF*2 at 100% dose) were 93% and 75% in the Rich- and Poor-LIPA groups, respectively. The proportion of patients with reduced treatment intensity was 2.4% and 10.7% in each group, while patients with enhanced treatment intensity were 4.9% and 14%. Anorexia at the first NAC was present in 56% and 71% in the Rich- and Poor-LIPA groups and 48% and 63% at the second and subsequent NACs. Although malnutrition before NAC was 41% and 21% in each group, it was 58% and 70% before the second course and 49% and 68% after the completion of NAC. Rates of dysphagia were not significantly different between the two groups. There were two patients (4.9%) in the Rich-LIPA group and three patients (11%) in the Poor-LIPA group who received tube feeding before NAC (*p* = 0.389) (Table 5).

**Table 5:** Clinical characteristics during neoadjuvant chemotherapy, surgical information, and postoperative clinical outcomes

Characteristic		Overall N = 69	Rich-LIPA^a^ N = 41	Poor-LIPA^b^ N = 28	*p* value
Clinical characteristics during neoadjuvant chemotherapy					
NAC regimen					0.078
Standard treatment (DCF*2 100% dose)		59 (86%)	38 (93%)	21 (75%)	
Reduced treatment intensity		4 (5.8%)	1 (2.4%)	3 (10.7%)	
DCF*1 100% dose		2 (2.9%)	1 (2.4%)	1 (3.6%)	
DCF*2 80% dose		2 (2.9%)	0 (0%)	2 (7.1%)	
Enhanced treatment intensity (DCF*3 100% dose)		6 (8.7%)	2 (4.9%)	4 (14%)	
Adverse events					
Leukopenia	1st^f^	48 (70%)	30 (73%)	18 (64%)	0.431
	2nd-^g^	26 (39%)	14 (35%)	12 (44%)	0.436
Febrile neutropenia	1st	21 (30%)	15 (37%)	6 (21%)	0.179
	2nd-	6 (9%)	3 (7.5%)	3 (11%)	0.679
Diarrhea	1st	36 (52%)	19 (46%)	17 (61%)	0.241
	2nd-	18 (27%)	9 (23%)	9 (33%)	0.326
Oral mucositis	1st	15 (22%)	11 (27%)	4 (14%)	0.215
	2nd-	8 (12%)	6 (15%)	2 (7.4%)	0.459
Anorexia	1st	43 (62%)	23 (56%)	20 (71%)	0.197
	2nd-	36 (54%)	19 (48%)	17 (63%)	0.213
Malaise	1st	55 (80%)	34 (83%)	21 (75%)	0.421
	2nd-	34 (51%)	20 (50%)	14 (52%)	0.882
Nutritional indexes					
Malnutrition^c^	T0^h^	23 (33%)	17 (41%)	6 (21%)	0.083
	T1^i^	45 (67%)	23 (58%)	19 (70%)	0.285
	T2^j^	39 (57%)	20 (49%)	19 (68%)	0.116
Dysphagia^d^	T0	40 (58%)	23 (56%)	17 (61%)	0.703
	T1	19 (28%)	10 (25%)	9 (33%)	0.458
	T2	9 (13%)	5 (12%)	4 (14%)	> 0.999
Tube feeding	T0	5 (7.2%)	2 (4.9%)	3 (11%)	0.389
	T1	2 (3.0%)	1 (2.5%)	1 (3.7%)	> 0.999
	T2	1 (1.4%)	1 (2.4%)	0 (0%)	> 0.999
Systemic inflammation indexes					
C-reactive protein (mg/dl)	T0	0.2 (0.1, 0.4)	0.2 (0.1, 0.4)	0.2 (0.1, 0.4)	0.942
	T2	0.1 (0.1, 0.3)	0.1 (0.1, 0.3)	0.1 (0.1, 0.2)	0.595
	Δ	−0.03 (−0.18, 0.02)	−0.03 (−0.13, 0.02)	−0.04 (−0.19, 0.04)	0.942
NLR^e^	T0	2.6 (1.8, 3.8)	2.2 (1.7, 3.5)	2.7 (2.1, 3.9)	0.325
	T2	1.9 (1.3, 2.7)	2.0 (1.2, 2.8)	1.9 (1.6, 2.4)	0.956
	Δ	−0.66 (−1.43, 0.14)	−0.55 (−1.01, 0.24)	−0.90 (−1.68, −0.12)	0.148
Surgical information					
Surgery time (min)		631 (554, 688)	606 (535, 685)	654 (568, 712)	0.187
Bleeding (ml)		158 (99, 240)	140 (94, 204)	170 (109, 273)	0.347
R0^k^		67 (97%)	41 (100%)	26 (93%)	0.161
Postoperative clinical outcomes					
Pneumonia		10 (14%)	5 (12%)	5 (18%)	0.729
Suture failure		3 (4.3%)	2 (4.9%)	1 (3.6%)	> 0.999
Surgical site infection		3 (4.3%)	2 (4.9%)	1 (3.6%)	> 0.999
ICU stay (days)^l^		4.0 (4.0, 5.0)	4.0 (4.0, 5.0)	4.0 (4.0, 5.0)	0.544
Postoperative days to walking (days)		3.0 (2.0, 4.0)	3.0 (2.0, 3.0)	3.0 (2.0, 4.0)	0.323
Adjuvant therapy		16 (23%)	6 (15%)	10 (36%)	0.042*

n (%); median (IQR)

Fisher's exact test; Wilcoxon rank sum test; Pearson's Chi-squared test

*LIPA* light intensity physical activity

*Significant difference (p < 0.05)

^a^Rich-LIPA patients whose LIPA in the treatment phase was ≥ 156.11 min/day

^b^Poor-LIPA patients whose LIPA in the treatment phase was < 156.11 min/day

^c^Malnutrition GNRI (geriatric nutritional risk index) score < 98

^d^Dysphagia difficulty eating a normal meal and requiring an adjusted diet or feeding tube

^e^NLR neutrophil-to-lymphocyte ratio (neutrophils/lymphocytes)

^f^1st 1st course of neoadjuvant chemotherapy

^g^2nd- 2nd or 3rd course of neoadjuvant chemotherapy

^h^T0 pre neoadjuvant chemotherapy

^i^T1 post 1st course of neoadjuvant chemotherapy

^j^T2 post neoadjuvant chemotherapy

^k^R0 no residual tumor

^l^ICU intensive care unit

### Surgical characteristics and postoperative clinical outcomes of the Rich- and Poor-LIPA groups

Although there was no significant difference, surgery time and bleeding tended to be reduced in the Rich-LIPA group. The R0 rate was 100% for the Rich-LIPA group compared to 93% for the Poor-LIPA group. A total of 15% and 36% of the patients in the Rich- and Poor-LIPA groups, respectively, required adjuvant therapy (Table 5).

## Discussion

### Summary of the main results

This study aimed to examine the impact of LIPA on tumor regression in male patients with esophageal cancer receiving NAC. The results suggest that increased LIPA is associated with tumor regression. Specifically, every extra 30-min increase in LIPA during the treatment phase was associated with tumor regression independent of age, body mass index, clinical stage at diagnosis, comorbidity index, wearing time of the accelerometer, and regimen (adjusted OR:1.41 [1.02–2.04]). Moreover, the cutoff value was 156.11 min/day, and patients with rich LIPA (≥ 156.11 min/day) were less likely to suffer from anorexia and malnutrition during NAC.

### Comparison to previous studies

To our knowledge, this is the first study to examine the impact of LIPA on tumor regression. Although several studies have demonstrated the effects of exercise on tumor regression and prognosis [6–8, 24], the optimal exercise program modality is still unclear. This lacking information was identified as a priority for research in the international Delphi study by the expert panel, patients, and their carers [25, 26]. This study contributed to overcoming the previous research gap by proposing a new exercise program, namely, enhancing LIPA. Patients with esophageal or gastroesophageal junction cancer undergoing NAC often experienced medical problems such as malnutrition [27] and psychiatric disorders [28] due to treatment toxicity. Consequently, exercise, based on the general recommendation of guidelines, is not easy for patients with esophageal cancer receiving NAC [9, 29]. Therefore, there is a need to develop a feasible physical activity program specifically for patients receiving NAC. We believe that LIPA, which is expected to have a high patient adherence rate [10], has a potential of promoting tumor regression. This is an important finding that will contribute to the supportive care of patients with esophageal cancer during NAC.

### Interpretation of the results

This study demonstrated that LIPA may impact tumor regression in male patients with esophageal cancer in the treatment phase of NAC. There are two hypothesized mechanisms through which LIPA may have contributed to tumor regression during the treatment period. First, LIPA may accelerate drug perfusion [30, 31]. Considering that LIPA in the intermission phase had no effect on tumor regression, it may be important to consider LIPA when the blood drug concentrations are elevated. Second, rich LIPA may have contributed to improved nutritional status and enhanced tumor immunity. Cachexia, characterized by cancer-related malnutrition, leads to tumor immune dysfunction [32]. Our results showed that the clinical characteristics of the Rich-LIPA group showed significantly higher LIPA levels in all courses, along with a lower incidence of anorexia and a tendency to maintain good nutritional status. While there was no significant difference between the two groups in dysphagia improvement after treatment. These findings suggest that rich LIPA in each course may have contributed to the alleviation of anorexia and the maintenance of nutritional status. Given that maintaining nutritional status is essential in pathologic tumor regression, LIPA may have contributed to tumor regression. Furthermore, the lack of significant improvement in dysphagia between the groups suggests that the maintenance of nutritional status in the Rich-LIPA group may have occurred independently of the clinical antitumor effects of chemotherapy. These hypotheses were supported by a multivariate analysis adjusted for the treatment regimen. Our clinical mechanisms remain hypothetical and require further investigation to elucidate the complex interplay between antitumor effects, anorexia, LIPA, and nutritional status.

In this study, LIPA was assessed using accelerometers with sufficient reliability, and its impact was evaluated using definitions of tumor regression that have a prognostic impact [23]. Moreover, we carefully examined the impact on LIPA, considering regimens and other confounding factors. This research methodology may have contributed to the quality assurance of the results of this study. Furthermore, the Rich-LIPA group demonstrated significant tumor regression based on computed tomographic images. This result supports an association between pathologic tumor regression and LIPA. Sensitivity analysis showed an association between LIPA and tumor regression (grade 2 ≤) (adjusted OR 1.24). This association may be weak compared to the association between LIPA and tumor regression defined as grade 1b or higher (adjusted OR 1.41).

### Implication of the results

The results highlight the importance of including rich LIPA as supportive care for patients with esophageal cancer during NAC. Interestingly, LIPA was associated with tumor regression, and the Rich-LIPA group showed favorable trends in operative time, blood loss, and R0 surgery. Rich LIPA may have contributed to the minimally invasive surgery through tumor regression. Furthermore, although not significant, rich LIPA also tended to promote clinical downstaging of lymph node metastases, which may have contributed to minimally invasive surgery. Because LIPA is highly feasible [10], our findings demonstrate the potential of a practical and beneficial physical activity program for patients with esophageal cancer during NAC. However, the results of this study generated a hypothesis but did not examine the effect of LIPA on tumor regression. In the future, a physical activity program including LIPA during NAC should be developed, validated in clinical trials, and applied clinically. According to the Physical Activity Compendium (https://pacompendium.com/), which has been widely used in research over the past 30 years, actual LIPA refers to physical activities that do not significantly increase the respiratory rate, such as slow walking, light stretching, yoga, grooming activities, standing work, watering plants, and other light daily tasks. A physical activity program with LIPA during the NAC will include these activities. Furthermore, although actual data showed no significant difference, the Poor-LIPA group tended to have more cases of tube feeding than the Rich-LIPA group (11% vs 4.9%). Therefore, patients with tube feeding should be considered for individualized interventions to prevent inactivity.

### Limitations

This study had several limitations. First, the sample size was small, and the study was conducted at a single center, which merits re-examination using a larger sample from multiple centers. Second, selection bias is possible, as only a subset of patients who had high compliance with prehabilitation during NAC or had the physical capacity to engage in 156 min of exercise per day were included in this study. Even among Stage III/IV patients, this activity level may not be realistic for those in more critical condition. Therefore, considering the results are specific to this limited patient group, additional scientific data is needed to determine whether LIPA is genuinely associated with tumor regression across a broader population. Moreover, patients with severe symptoms may have trouble wearing and managing activity trackers. Therefore, it may be essential to develop an activity assessment system that is manageable even for such groups. Third, this was a retrospective observational study, and the effect of LIPA on tumor regression, namely causal inference, is unclear. Fourth, 90% of the patients were diagnosed with SCC, which requires re-examination of patients diagnosed with adenocarcinomas. Fifth, male patients were exclusively included to eliminate the influence of gender differences on the relationship between LIPA and tumor regression. The previous study [6] suggested that physical activity may have similar effects regardless of gender. However, the results obtained from male patients with esophageal cancer cannot yet be generalized to female patients, and further research is required. Finally, the long-term outcomes of patients remain unknown. Prehabilitation programs that include LIPA are required to be developed and their effectiveness should be examined in clinical trials.

## Conclusions

This study demonstrated that LIPA during NAC has a potential of promoting tumor regression with a cutoff value of 156.5 min/day. Further clinical research is required to determine the prognostic benefits of LIPA in patients receiving NAC.

## Supplementary Information

Below is the link to the electronic supplementary material.

Supplementary file1 (DOCX 28 KB)
