# Supplementary material for: Association between accelerometer-measured light-intensity physical activity and tumor regression for male patients with esophageal cancer receiving neoadjuvant therapy: a retrospective cohort study
Source: Esophagus. 2025 Feb 4;22(2):177–87. doi: 10.1007/s10388-025-01108-9 (PMC11929633; doi:10.1007/s10388-025-01108-9)
Supplement: Supplementary file 1 — Supplementary file1 (DOCX 28 KB) [file 10388_2025_1108_MOESM1_ESM.docx]

**Online Resource**

**Journal: Esophagus**

**Association Between Accelerometer-measured Light-intensity Physical Activity and Tumor Regression for Male Patients with Esophageal Cancer Receiving Neoadjuvant Therapy: A Retrospective Cohort Study**

Tomohiro Ikeda, MSc^1^, Kazuhiro Noma, MD, PhD^2,3*^, Masanori Konuma, BSc^1^, Naoaki Maeda, MD, PhD^2,3^, Shunsuke Tanabe, MD, PhD^2,3^, Takayoshi Kawabata, BSc^4^, Masashi Kanai, PhD^5^, Masanori Hamada, MD, PhD^1^, Toshiyoshi Fujiwara, MD, PhD^2,3^, Toshifumi Ozaki, MD, PhD^1,6^

^1^Department of Rehabilitation Medicine, Okayama University Hospital, 2-5-1 Shikatacho, Kita-ku, Okayama 700-8558, Japan

^2^Department of Gastroenterological Surgery, Graduate School of Medicine, Dentistry and Pharmaceutical Sciences, Okayama University, 2-5-1 Shikatacho, Kita-ku, Okayama 700-8558, Japan

^3^Center for Esophageal Disease, Okayama University Hospital, 2-5-1 Shikatacho, Kita-ku, Okayama 700-8558, Japan

^4^Department of Pharmacy, Okayama University Hospital, 2-5-1 Shikatacho, Kita-ku, Okayama 700-8558, Japan

^5^Institute of Transdisciplinary Sciences for Innovation, Kanazawa University, Ishikawa, Japan

^6^Department of Orthopaedic Surgery, Okayama University Hospital, 2-5-1 Shikatacho, Kitaku, Okayama 700-8558, Japan

**^*^Corresponding author:** Kazuhiro Noma

Department of Gastroenterological Surgery, Graduate School of Medicine, Dentistry and Pharmaceutical Sciences, Okayama University 2-5-1 Shikatacho, Kita-ku, Okayama 700-8558, Japan

E-mail address: knoma@md.okayama-u.ac.jp

Telephone number: 81-86-235-7752; Fax number: 81-86-235-7752

Online Resource 1. Impact of every extra 30-minutes of light intensity physical activity on the tumor regression (grade 2≤)

|  | Crude Model^a^ |  |  | Adjusted Model 1^b^ |  |  | Adjusted Model 2^c^ |  |
| --- | --- | --- | --- | --- | --- | --- | --- | --- |
|  | OR^d^ (95%CI^e^) | p-value |  | OR (95%CI) | p-value |  | OR (95%CI) | p-value |
| TR-LIPA^f^ (30 min/d) | 1.28 (0.97, 1.73) | 0.092 |  | 1.25 (0.94, 1.70) | 0.137 |  | 1.24 (0.93, 1.70) | 0.149 |
| INT-LIPA^g^ (30 min/d) | 0.93 (0.75, 1.12) | 0.452 |  | 0.86 (0.66, 1.08) | 0.270 |  | 0.83 (0.63, 1.06) | 0.154 |
| The results of regression analysis are shown for the impact of light intensity physical activity measured by accelerometer on tumor regression (grade 2≤: characterized by necrosis or fibrosis in more than 2/3).  *Indicates statistically significant difference  ^a^**Crude Model** univariate analysis  ^b^**Adjusted Model** **1** adjusted for age, BMI, stage, CCI, and wearing time of the accelerometer  ^c^**Adjusted Model** **2** adjusted for age, BMI, stage, CCI, wearing time of the accelerometer, and regimen (standard, reduced or enhanced)  ^d^**OR** odds ratio; ^e^95% CI 95% confidence interval  ^f^**TR-LIPA** light intensity physical activity in the treatment phase  ^g^**INT-LIPA** light intensity physical activity in the intermission phase | | | | | | | | |

Online Resource 2. Impact of light intensity physical activity on the tumor regression (Grade 2≤)

|  | Crude Model^a^ |  |  | Adjusted Model 1^b^ |  |  | Adjusted Model 2^c^ |  |
| --- | --- | --- | --- | --- | --- | --- | --- | --- |
|  | OR^d^ (95%CI^e^) | p-value |  | OR (95%CI) | p-value |  | OR (95%CI) | p-value |
| Rich-LIPA (TR)^f^ | 5.33 (1.80-18.4) | 0.004* |  | 4.96 (1.64-17.4) | 0.007* |  | 5.38 (1.69-20.1) | 0.007* |
| Rich-LIPA (INT)^g^ | 1.12 (0.39-3.36) | 0.836 |  | 0.90 (0.27-2.99) | 0.864 |  | 0.77 (0.22-2.63) | 0.671 |
| The results of regression analysis are shown for the impact of light intensity physical activity measured by accelerometer on tumor regression (grade 2≤: characterized by necrosis or fibrosis in more than 2/3).  *Indicates statistically significant difference  ^a^**Crude Model**: univariate analysis  ^b^**Adjusted Model** **1** adjusted for age, BMI, stage, CCI, and wearing time of the accelerometer  ^c^**Adjusted Model** **2** adjusted for age, BMI, stage, CCI, wearing time of the accelerometer, and regimen (standard, reduced or enhanced)  ^d^**OR** odds ratio; **^e^95% CI** 95% confidence interval  ^f^**Rich-LIPA in TR** patients whose light intensity physical activity in the treatment phase was >156.1 min  ^g^**Rich-LIPA in IN** patients whose light intensity physical activity in the intermission phase was > 191.3min | | | | | | | | |

Online Resource 3. STROBE Statement—Checklist of items that should be included in reports of *cohort studies*

|  | Item No | Recommendation | Page |
| --- | --- | --- | --- |
| **Title and abstract** | 1 | (*a*) Indicate the study’s design with a commonly used term in the title or the abstract | P1 |
|  |  | (*b*) Provide in the abstract an informative and balanced summary of what was done and what was found | P3 |
| Introduction | | |  |
| Background/rationale | 2 | Explain the scientific background and rationale for the investigation being reported | P4 |
| Objectives | 3 | State specific objectives, including any prespecified hypotheses | P4 |
| Methods | | |  |
| Study design | 4 | Present key elements of study design early in the paper | P5 |
| Setting | 5 | Describe the setting, locations, and relevant dates, including periods of recruitment, exposure, follow-up, and data collection | P5 |
| Participants | 6 | (*a*) Give the eligibility criteria, and the sources and methods of selection of participants. Describe methods of follow-up | P5 |
|  |  | (*b*) For matched studies, give matching criteria and number of exposed and unexposed | Not applicable |
| Variables | 7 | Clearly define all outcomes, exposures, predictors, potential confounders, and effect modifiers. Give diagnostic criteria, if applicable | P5-6 |
| Data sources/ measurement | 8* | For each variable of interest, give sources of data and details of methods of assessment (measurement). Describe comparability of assessment methods if there is more than one group | P5-6 |
| Bias | 9 | Describe any efforts to address potential sources of bias | P6-7 |
|  |  |  |  |
| Study size | 10 | Explain how the study size was arrived at | P5 |
| Quantitative variables | 11 | Explain how quantitative variables were handled in the analyses. If applicable, describe which groupings were chosen and why | Not applicable |
| Statistical methods | 12 | (*a*) Describe all statistical methods, including those used to control for confounding | P6-7 |
|  |  | (*b*) Describe any methods used to examine subgroups and interactions | Not applicable |
|  |  | (*c*) Explain how missing data were addressed | P5 |
|  |  | (*d*) If applicable, explain how loss to follow-up was addressed | P5 |
|  |  | (*e*) Describe any sensitivity analyses | P7 |
| Results | | |  |
| Participants | 13* | (a) Report numbers of individuals at each stage of study—eg numbers potentially eligible, examined for eligibility, confirmed eligible, included in the study, completing follow-up, and analysed | P8 |
|  |  | (b) Give reasons for non-participation at each stage | P8 |
|  |  | (c) Consider use of a flow diagram | Appendix S1 |
| Descriptive data | 14* | (a) Give characteristics of study participants (eg demographic, clinical, social) and information on exposures and potential confounders | P8 |
|  |  | (b) Indicate number of participants with missing data for each variable of interest | P8 |
|  |  | (c) Summarise follow-up time (eg, average and total amount) | Not applicable |
| Outcome data | 15* | Report numbers of outcome events or summary measures over time | P8-9 |
| Main results | 16 | (*a*) Give unadjusted estimates and, if applicable, confounder-adjusted estimates and their precision (eg, 95% confidence interval). Make clear which confounders were adjusted for and why they were included | P8-9 |
|  |  | (*b*) Report category boundaries when continuous variables were categorized | P8-9 |
|  |  | (*c*) If relevant, consider translating estimates of relative risk into absolute risk for a meaningful time period | Not applicable |
| Other analyses | 17 | Report other analyses done—eg analyses of subgroups and interactions, and sensitivity analyses | P9 |
| Discussion | | |  |
| Key results | 18 | Summarise key results with reference to study objectives | P11 |
| Limitations | 19 | Discuss limitations of the study, taking into account sources of potential bias or imprecision. Discuss both direction and magnitude of any potential bias | P13 |
| Interpretation | 20 | Give a cautious overall interpretation of results considering objectives, limitations, multiplicity of analyses, results from similar studies, and other relevant evidence | P11-12 |
| Generalisability | 21 | Discuss the generalisability (external validity) of the study results | P12-13 |
| Other information | | |  |
| Funding | 22 | Give the source of funding and the role of the funders for the present study and, if applicable, for the original study on which the present article is based | P2 |

*Give information separately for exposed and unexposed groups.

**Note:** An Explanation and Elaboration article discusses each checklist item and gives methodological background and published examples of transparent reporting. The STROBE checklist is best used in conjunction with this article (freely available on the Web sites of PLoS Medicine at http://www.plosmedicine.org/, Annals of Internal Medicine at http://www.annals.org/, and Epidemiology at http://www.epidem.com/). Information on the STROBE Initiative is available at http://www.strobe-statement.org.
